# Assessment of a University Campus Food Environment, California, 2015

**DOI:** 10.5888/pcd13.150455

**Published:** 2016-02-04

**Authors:** Marilyn Tseng, Kelsey DeGreef, Madison Fishler, Rachel Gipson, Kelly Koyano, Dawn B. Neill

**Affiliations:** Author Affiliations: Kelsey DeGreef, Madison Fishler, Rachel Gipson, Kelly Koyano, Dawn B. Neill, California Polytechnic State University, San Luis Obispo, California.

## Abstract

**Introduction:**

University campuses offer an opportunity to study the extent to which modifying the food environment influences eating, but in-depth characterizations of campus food environments are needed to identify potential targets for intervention. The objective of this project was to describe the availability, accessibility, and quality of healthful food choices in dining venues and food stores at or near a public, 4-year university in California.

**Methods:**

Trained assessors used the Nutrition Environment Measures Survey for campus dining (NEMS-CD) to evaluate all 18 campus dining venues, and NEMS for stores (NEMS-S) to evaluate 2 on-campus and 37 off-campus food stores. We calculated prevalence of healthful and unhealthful constructs (eg, availability of selected food items, presence of signage encouraging healthful eating, pricing options that encourage healthful eating), based on the NEMS and compared scores across different types of venues.

**Results:**

NEMS-CD scores ranged from 4 to 47 (mean [SD], 26.0 [14.4]) out of a possible maximum score of 97; 12% of entrées and 36% of main dish salads served in these venues were classified as healthful. NEMS-S score for the 2 on-campus food stores (24 for both) was intermediate between off-campus convenience stores (mean [SD], 12.0 [5.3]) and grocery/supermarket stores (mean [SD], 31.1 [10.0]), with a possible maximum score of 54.

**Conclusion:**

Standardized environmental evaluation provides insights into both positive and negative aspects of campus community food venues. Environmental assessment identifies potential targets for modification and baseline data for designing and implementing action-oriented research aimed at improving the campus food environment’s support of healthful food choices for college students.

## Introduction

University campuses are an informative setting in which to describe the effect of the food environment on individual food choices. At many 4-year colleges and universities in the United States, first-year students eat most if not all of their main meals on campus, and on-campus venues serve a substantial proportion of students even if they live off campus. However, evidence suggests that the food available on campus is not conducive to healthful eating (1,2). A study of 15 postsecondary institutions concluded that “the full campus dining environment provides limited support for healthful eating and obesity prevention” (3), and weight gain among first-year college students has been well documented (4–8).

Where to target interventions in the campus food environment is not clear. Interventions could target specific venues — for example, fast food restaurants, which are linked to poor diet, overweight and obesity (9), and metabolic outcomes (10) among young adults — or more general policies — for example, providing on-site nutrition information (11–13). An assessment of a campus food environment adds to knowledge regarding the quality of the food available at different venues in a college setting (3,14). It can also serve as a basis for recommending modifications to the food environment.

We used the Nutrition Environment Measures Survey (NEMS) to evaluate the food environment at a public, 4-year university in the Central Coast region of California. We assessed all dining venues and the 2 food stores on campus as well as all food stores in the surrounding area as a basis for comparison with the 2 campus food stores.

## Methods

The California Polytechnic State University (Cal Poly), a public institution with approximately 19,000 undergraduates (15), is in San Luis Obispo, a city on the Central Coast of California with a population of about 45,000. A higher proportion of Cal Poly’s undergraduates live on campus than students at other public, 4-year institutions (37% vs 23%), including 98% of its first-year students (15,16). At the time of data collection, the campus had 2 food markets and 18 dining venues, including fast food and brand-name stores, food courts, and restaurant-style and cafeteria-style venues, following a trend toward a high-variety food environment now common at universities (17).

### Enumeration

We identified 18 dining venues and 2 stores from the university web site (18). We used NEMS definitions to categorize dining venues as sit-down (n = 1, full table service by wait staff), fast food (n = 6, minimal service, food cooked in bulk in advance and kept hot or reheated to order, then supplied quickly after ordering), fast casual (n = 3, food ordered and paid for at a counter but brought to table, offering higher quality of food and atmosphere than at fast food), or specialty shops offering primarily nonentrée items (n = 5, eg, coffee, pastries, yogurt, smoothies). In addition, we treated separately 2 campus venues that are conglomerations of fast food establishments in 1 area as food courts. The 1 campus venue offering all-you-can-eat pricing was also analyzed separately.

We identified 51 off-campus food stores within the city of San Luis Obispo by using a combination of Internet sources (Google.com, yellowpages.com, Yelp.com), as well as local knowledge of the city. We found an additional 28 businesses listed as food stores in the complete list of businesses with active business licenses through November 2014, available on the City of San Luis Obispo website (19). Stores were categorized as grocery/supermarket, pharmacy, gas station convenience store, or other convenience store. We excluded stores requiring membership and specialty stores such as liquor stores, ethnic food markets, and bakeries, leaving a total of 41 stores including the 2 on campus. In April 2015, two supermarkets closed and re-opened under new ownership and management; these were added to the list. Of the 43 stores, 1 was no longer in business and another was closed for renovation on the day of assessment; a third declined assessment; and we were unable to evaluate 1 of the newly opened stores within our data collection period, leaving a total of 39 stores assessed for the project.

### Measures

We used the NEMS, which focuses on the availability, affordability, and quality of healthful food choices, to evaluate the campus food environment. The NEMS is a widely used instrument with demonstrated inter-rater and test–retest reliability (20,21). It has been used in various settings as a basis for improving food environments and access to healthful food choices, and in research to describe differences in nutrition environments across socioeconomically disparate neighborhoods (22–25). Detailed information on NEMS standard procedures for data collection and scoring, as well as information on its development, reliability, and applications in research and practice, are published (20,21) or available online (26).

We used NEMS for campus dining (NEMS-CD) (3) to evaluate the 18 campus dining venues, and NEMS for stores (NEMS-S) to evaluate the 39 food stores. NEMS-S assesses availability of healthful food choices, healthful food pricing, and quality of produce in food stores (20). NEMS-CD was adapted from the NEMS for restaurants (NEMS-R), which assesses facilitators of and barriers to healthful eating (eg, pricing; availability of nutrition information; portion sizes; availability of healthful entrées, fruits, vegetables, and beverages). NEMS-CD includes additional items specific to a campus environment, including salad bar quality and dining contracts (3).

### Data collection

Four student investigators were trained and certified to use standard procedures for applying the NEMS instruments. After training, each investigator individually conducted at least 2 practice audits of different store (supermarket, gas station convenience store) or restaurant (fast food, fast casual) types until there was more than 85% agreement between investigators’ assessments.

All data were collected between March and August 2015. Two student investigators evaluated campus dining venues using NEMS-CD, which included visiting each food venue between 11 AM and 2 PM on a weekday and reviewing Internet and menu information for each venue. After evaluating each venue, they discussed any unclear items and completed the instrument by consensus. They followed the same procedures to conduct repeat evaluations of 9 of the 18 venues to assess test–retest reliability (mean, 59 days between assessments; range, 42–77 days). The intra-class correlation coefficient was 0.93 (95% CI, 0.76–0.98). Of 44 constructs (eg, availability of selected food items, presence of signage encouraging healthful eating, pricing options that encourage healthful eating) considered in scoring, mean percentage agreement between assessments was 89.9% (range, 77.3%–97.7%).

The other 2 student investigators were each assigned approximately 20 stores to evaluate using NEMS-S. Five of the stores were evaluated twice to assess inter-rater reliability (mean, 23.8 days between assessments; range, 1–68 days). Intra-class correlation coefficient was 0.96 (95% CI, 0.77–0.99). Of 28 constructs considered in scoring, mean percentage agreement between assessments was 92.9% (range, 85.7%–100%).

### Scoring

NEMS-CD scores were based on 6 subscores (3) (T.M. Horacek, PhD, RD, written communication, February 2015):

1. Healthy entrées and main dish salads (range, 0–9), where “healthy” was defined according to NEMS procedures as having a) ≤800 kcals (≤650 kcals for a la carte burgers and sandwiches), b) ≤30% of calories from fat, AND c) ≤10% of calories from saturated fat.
2. Healthy side dishes (fruits without sugar, whole grain items, baked chips, nonfried vegetables, healthy cereals, and salad bar; range, 0–35).
3. Healthy beverages (diet soda, 100% fruit juices, low-fat or skim milk, milk alternatives, other low-calorie beverages; range, 0–18).
4. Healthy eating facilitators (based on availability of nutrition information and signage; range, 0–30).
5. Healthy eating barriers (based primarily on signage; range −15 to 0).
6. Pricing (charging more for healthy items, encouraging large portions, or discouraging small portions; range, −14 to 5).

An overall score with a possible range from −29 to 97 points was calculated for each dining venue as the sum of the 6 subscores. Higher scores indicate greater availability of healthful options, more facilitators or fewer barriers to healthful eating, or pricing that encourages healthful eating, or any combination of these factors. One item from Horacek et al’s original subscore for entrées and main dish salads, on whether special requests were taken, was omitted from our assessment, resulting in a maximum entrées and main dish salads subscore of 9 rather than 12, and a maximum possible overall score of 97 rather than 100.

NEMS-S scores were based on 3 subscores (20):

1. Availability of healthful options in 11 food categories (low-fat or skim milk, fresh fruit, fresh vegetables, lean ground beef, fat-free or reduced-fat hot dogs, reduced-calorie frozen dinners, low-fat baked goods, diet sodas and 100% fruit juices, whole-grain bread, baked chips, and low-sugar cereal; range, 0–30).
2. Pricing of healthful versus regular options (range, −9 to 18).
3. Produce quality (range, 0–6).

An overall score with a possible range from −9 to 54 points was calculated for each store as the sum of the 3 subscores. Higher scores indicate greater availability of healthful items, lower pricing for healthful items, better quality produce, or a combination of these factors.

### Statistical analyses

We determined the distribution of categories for each construct and calculated subscores and total scores. All statistical analyses were conducted using SAS (version 9.4, SAS Institute, Inc).

## Results

### Campus dining venues

Healthful entrées were available in 9 of the 18 campus dining venues, and healthful main dish salads in 6 (Table 1). In total, 36 (12%) of 314 entrées and 11 (36%) of 31 main dish salads served in these venues were classified as healthful. The most commonly available side dish items were fruits without sugar, whole grain items, and nonfried vegetables. Diet soda was the most readily available low-calorie beverage. While most venues provided nutrition information on the Internet, few provided information to facilitate healthful choices on site: 9 provided nutrition information on menus, 8 had signs highlighting healthful menu items, and 6 had nutrition information at the point of purchase. Similar proportions of venues had signs encouraging healthful eating as had signs encouraging unhealthful eating, and 3 had signs encouraging both. Overall, small portions were not priced higher than large portions, but 2 coffee shops charged more for the more healthful than for the regular option (Table 1).

**Table 1:** Dining Venues^a^ With Availability of Selected Constructs and Mean Scores and Subscores From the Nutrition Environment Measures Survey for Campus Dining (NEMS-CD), California Polytechnic State University at San Luis Obispo, March–August 2015

Construct	All Venues (n = 18)	Fast Food (n = 6)	Fast Casual (n = 3)	Food Court (n = 2)	All-You-Can-Eat (n = 1)	Sit-Down (n = 1)	Specialty (n = 5)
**Venues with selected construct, %**							
**Entrées**							
Healthful^b^ entrees	50	50	67	100	100	100	0
Healthful^b^ main dish salads	33	17	100	50	0	0	20
**Side dishes**							
Fruits without sugar	61	33	100	100	100	0	60
Whole grain items	44	50	100	50	0	100	0
Nonfried vegetables	39	33	33	100	100	0	20
Baked chips	28	33	33	100	0	0	0
Healthful cereals	28	33	0	50	100	0	20
Salad bar	22	14	33	100		0	0
**Beverages**							
Diet soda	56	67	67	100	100	100	0
100% fruit juice	50	50	33	100	0	0	60
Low-fat or skim milk	35	33	33	100	0	0	80
**Healthful eating facilitators**							
Nutrition information available via Internet	94	100	100	100	100	0	100
Menu has nutrition information or healthy items labeled	50	50	33	0	100	0	80
Signs highlight healthful menu items	44	67	67	0	0	0	40
Signs encourage healthful eating	33	33	33	50	0	0	40
Nutrition information available at point of purchase	33	33	33	0	0	0	60
Healthful requests encouraged	11	17	33	0	0	0	0
**Healthful eating barriers**							
Signs encourage unhealthful eating	28	17	33	50	0	100	20
Large portions encouraged	22	0	33	0	100	100	20
Signs encourage overeating	11	17	33	0	0	0	0
Signs discourage special requests	11	0	0	0	0	100	20
All-you-can-eat or unlimited trips	6	0	0	0	100	0	0
**Pricing**							
Combo meal cheaper than individual items	6	17	0	0	0	0	0
Reduced portion sizes offered	0	0	0	0	0	0	0
Healthful items costlier than regular	11	0	0	0	0	0	40
Charge for shared entrée	0	0	0	0	0	0	0
Smaller portion cheaper than regular	0	0	0	0	0	0	0
**Score, mean (SD)**							
**NEMS-CD (−29 to 97)**	26.0 (14.4)	25.7 (17.6)	29.7 (16.5)	44.0 (4.2)	29.0	5.0	20.6 (5.6)
**Subscores**							
Healthful entrée^b^ or main dish salad (range of possible scores, 0 to 9)	2.0 (2.4)	2.2 (3.5)	4.0 (1.7)	3.0 (0.0)	2.0	2.0	0.2 (0.4)
Healthful^b^ side dish (range of possible scores, 0 to 35)	7.5 (7.5)	4.3 (4.8)	11.3 (5.5)	21.5 (2.1)	17.0	4.0	2.2 (2.7)
Healthful beverage (range of possible scores, 0 to 18)	8.4 (5.7)	7.3 (5.9)	6.3 (5.8)	17.0 (0.0)	6.0	6.0	8.4 (5.9)
Healthful eating facilitators (range of possible scores, 0 to 30)	9.6 (6.9)	11.3 (10.3)	9.0 (4.6)	5.0 (0.0)	10.0	0	11.4 (4.2)
Healthful eating barriers (range of possible scores, −15 to 0)	−2.3 (3.0)	−1.0 (1.5)	−3.0 (5.2)	−1.5 (2.1)	−6.0	−9.0	−1.8 (1.6)
Pricing (range of possible scores, −14 to 5)	1.1 (1.4)	1.5 (1.2)	2.0 (0.0)	−1.0 (0.0)	0.0	2.0	0.8 (1.6)

a We used NEMS definitions to categorize dining venues as sit-down (full table service by wait staff), fast food (minimal service, food cooked in bulk in advance and kept hot or reheated to order, then supplied quickly after ordering), fast casual (food ordered and paid for at a counter but brought to table, offering higher quality of food and atmosphere than at fast food), or specialty shops offering primarily nonentrée items (eg, coffee, pastries, yogurt, smoothies). Food courts were campus venues that are conglomerations of fast food establishments in 1 area.

b Healthful defined as having 1) ≤800 kcals (≤650 kcals for a la carte burgers and sandwiches), 2) ≤30% of calories from fat, AND 3) ≤10% of calories from saturated fat.

Overall mean NEMS-CD score was 26.0 (SD, 14.4) and ranged from 4 to 47. The 2 food courts had among the highest scores (41 and 47), and the sit-down restaurant had the lowest (5). Higher scores in food courts were due to greater availability of healthful side dishes and beverages, although they offered fewer facilitators to healthful eating compared with most other venue types (Table 1).

Fast food venues showed the greatest range of overall scores (4 to 47). They tended to have lower scores than the fast casual restaurants or food courts with respect to healthful entrées and sides. On the other hand, 1 fast food restaurant had the highest score overall for healthful entrée availability (subscore 9), another had the highest score for healthful beverage availability (subscore 17), 2 had the highest scores overall for facilitators to healthful eating (subscore 24), and 4 had the fewest barriers to healthful eating (subscore 0). The one venue offering all-you-can-eat pricing had a high score overall due primarily to the range of healthful side dishes available. The low overall score for the sit-down restaurant was due to low subscores for healthful entrees, healthful sides, facilitators, and barriers (signs encouraging unhealthful choices or discouraging special requests). Not surprisingly, specialty shops were less likely than the other venues to offer healthful entrées and side dishes, but as a group they had the highest overall facilitators subscore.

### Campus food stores

NEMS-S scores for the 2 campus stores were intermediate between mean scores for grocery/supermarket stores and all other store types (Table 2). A wide variety of healthful food choices were available in the 2 campus stores, including low-fat milk, fresh produce of acceptable quality, diet soda, 100% fruit juice, whole-grain bread, baked chips, and low-sugar cereals. Absent in the campus stores were lean ground beef and low-fat baked goods. Fat-free or reduced-fat hot dogs and reduced-fat frozen dinners were available but with a limited selection. More healthful choices were not priced higher than regular versions, with the exception of baked chips. In contrast, pharmacies, gas station convenience stores, and other convenience stores had lower availability of healthful food choices compared with grocery/supermarket stores, which were most likely of all store types to charge lower prices for healthful food options than for regular options (data not shown).

**Table 2:** Distribution of Overall Scores, Subscores, and Selected Constructs for Nutrition Environment Measures Survey for Stores (NEMS-S) in Food Stores on the Campus of the California Polytechnic State University in San Luis Obispo, and in Off-Campus Food Stores in San Luis Obispo, California, March–August 2015

Construct	Campus Food Store (n = 2)	Grocery/Supermarket (n = 16)	Pharmacy (n = 5)	Gas Station Convenience Store (n = 13)	Other Convenience Store (n = 3)
**Score, mean (SD)**					
**NEMS-S (range of possible scores, −9 to 54)**	24.0^a^	31.1 (10.0)	12.4 (6.3)	6.8 (3.6)	12.0 (5.3)
**Subscores**					
Availability of healthful options (range of possible scores, 0 to 30)	16.0^a^	22.6 (7.1)	12.2 (3.3)	6.2 (3.6)	9.7 (2.1)
Pricing of healthful versus regular options (range of possible scores, −9 to 18)	2.0^a^	3.1 (4.0)	0.2 (3.3)	−0.3 (1.1)	−0.3 (3.8)
Produce quality (range of possible scores, 0 to 6)	6.0^a^	5.4 (1.6)	0 (0)	0.9 (1.4)	2.7 (0.6)
**Stores with selected construct, %**					
**Availability of healthful options**					
Low-fat milk or skim milk	100	94	80	31	33
No. of fresh fruit varieties					
1–5	0	19	0	23	100
>5	100	81	0	8	0
No. of fresh vegetable varieties					
1–5	50	6	0	0	0
>5	50	81	0	0	0
Lean ground beef	0	81	0	0	0
Fat-free or reduced-fat hot dogs	50	75	20	0	0
Reduced-fat frozen dinners	50	81	80	23	33
Low-fat baked goods	0	81	60	0	0
Diet soda	100	100	100	100	100
100% Fruit juice	100	94	100	85	100
Whole-grain bread	100	88	40	8	100
Baked chips	100	75	60	46	33
Low-sugar cereal	100	94	100	46	100
**Healthful option priced higher than regular option**					
Diet soda	0	0	0	0	0
Juice (vs juice drink)	0	56	40	8	33
Baked chips	100	69	60	46	33
Lowest-fat milk	0	0	20	15	33
Lean beef	0	69	0^b^	0^b^	0^b^
Fat-free or reduced-fat hot dogs	0	19	0	0^b^	0^b^
Reduced-calorie frozen dinners	0	13	20	8	33
Low-fat baked goods	0	13	20	0^b^	0^b^
Whole-grain bread	0	25	20	8	67
Low-sugar cereal	0	31	60	46	67
**Produce quality**					
≥75% fruits acceptable^c^	100	94	0^b^	31	67
≥75% vegetables acceptable^c^	100	88	0^b^	0^b^	0^b^

a Scores and subscores were the same for the 2 campus stores.

b Not available in any stores of this type.

c Proportion of different varieties of fruits or vegetables available at each store that were deemed to be of acceptable quality.

## Discussion

This study is unique in providing a complete picture of the food environment of 1 campus community. A primary finding was that campus dining venues showed substantial differences in the healthfulness of offerings. Though most venues offered some dishes that were classified as healthful entrées or main dish salads, most entrées and main dish salads available were not classified as healthful. In contrast to many assumptions, fast-food restaurants on campus did not have uniformly low scores, with a fast-food venue having the highest overall score for healthful entrée availability, and our findings produced low scores for the sit-down restaurant. Additionally, our findings showed that food courts offered much potential for availability of healthful choices largely by providing greater healthful side dish and beverage options. Finally, our findings indicate a greater role for campus food stores by offering more choice than typical convenience stores. In sum, these results provide insight into planning interventions to make the food environment more conducive to healthful eating by students.

Overall NEMS-CD score (26.0) for this campus was low relative to a maximum possible score of 97, but consistent with other comparably sized universities (15,001–29,999 students). In a study by Horacek et al (3), mean scores ranged from 20 to 46 depending on type of food venue. Compared with other similarly-sized institutions (3), the Cal Poly food environment scored higher with respect to healthful eating facilitators and pricing but less well with respect to healthful side dishes and low-calorie beverages. The 2 food courts compared favorably with those surveyed by Horacek et al (3), due primarily to greater availability of healthful side dishes. Campus fast food restaurants varied considerably in score, with the only sit-down restaurant having the lowest score overall. These findings are consistent with a comparison of 102 fast-food and 115 sit-down restaurants in 4 neighborhoods in Atlanta, Georgia, which showed neither type to be consistently more healthful than the other (21).

The 2 on-campus stores scored much higher than off-campus convenience stores and almost as well as grocery/supermarket stores overall, with lower points due primarily to less availability of certain healthful food items. In the 1 previous study that evaluated food stores on or near college campuses (14), grocery stores had higher availability of healthful food alternatives and fruits and vegetables than convenience stores, including on-campus stores (14). However, grocery stores were more likely than convenience stores to charge more for healthful foods and beverages (14). We found the opposite: grocery/supermarket stores were more likely than convenience stores to charge less for healthful foods and beverages; the 2 on-campus stores charged the same for healthful and regular alternatives for all items except low-sugar cereal. Notably, the 2 campus stores at Cal Poly offered produce of acceptable quality. Overall NEMS-S scores for the 2 campus stores indicate that they function more as grocery stores than as typical convenience stores by offering a greater variety of healthful food choices. Our findings provide a useful counterexample to the typical view of campus stores as convenience stores only. Such on-campus food stores may serve as a convenient source of food for students who choose to prepare their own basic meals.

This study is subject to at least 4 limitations worth discussion. First, daily fluctuations may have contributed to variability in scoring, although repeat assessments showed good test-retest reliability for NEMS-CD scoring. In stores, seasonal produce availability may also have contributed to variability in scoring, although we saw no pattern of difference in total NEMS-S score or availability subscore by month of assessment (data not shown). Second, while NEMS scoring provides a useful quantitative measure of aspects of a healthful food environment, it does not consider price differences across stores (ie, whether the same item is more expensive in one store than another). In addition, whether eating at a higher-scoring venue leads to more healthful eating was beyond the scope of the study. Lesser et al (27), for example, found that adolescents, although ordering more vegetables, still purchased the same amount of calories at Subway as at McDonald’s, concluding that, “despite [Subway’s] being marketed as ‘healthy,’ . . . meals from both restaurants are likely to contribute to overeating.” Other work, however, provides evidence that environmental modifications as simple as providing point-of-purchase nutritional information or positive signage can have a beneficial impact in college settings (11,12). Finally, the campus food environment described here may not reflect other campus food environments. Nevertheless, it serves as a useful case study for observing the diversity of venues available within a relatively small area. The findings highlight some positive aspects of this campus food environment: the high proportion of venues offering healthful entrées and the availability of nutrition information online. They also highlight areas for improvement, which can be the basis for future intervention. These include increasing the proportion of healthful entrées overall, improving the variety of healthful side dishes and beverages, making nutrition information available at point of purchase, and providing signage and implementing pricing strategies to facilitate more healthful eating choices.

In a study of college students living off-campus, more frequent purchasing of foods on and around campus was associated with poorer dietary habits, while bringing food from home was associated with healthier dietary patterns (1). For on-campus students, who are often required to purchase a dining plan and have limited access to a kitchen, almost every meal is equivalent to “eating out,” and eating out is consistently associated with poor diet quality (28). Given students’ necessary dependence on the campus food environment, universities have a responsibility to provide a food environment that facilitates and supports healthful eating, at a minimum by increasing the availability of healthful prepared foods or healthful foods to be prepared as meals. Information from this study will serve as baseline data to design an action research project toward improving Cal Poly’s food environment. Further research and evaluation will be needed to determine the extent to which a campus food environment is amenable to change given its constraints (29) and to determine the extent to which food environment modifications facilitate healthful food choices by students.
